# Mid-term follow-up results of neoadjuvant sintilimab combined with chemotherapy for locally advanced resectable esophageal squamous cell carcinoma

**DOI:** 10.3389/fimmu.2024.1453176

**Published:** 2024-12-04

**Authors:** Haibo Cai, Liji Chen, Junjun Huang, Hongmei Ma, Shifa Zhang, Kaize Zhong, Dongbao Yang, Jiuhe Sun, Hongfeng Liu, Ru Song

**Affiliations:** ^1^ Department of Thoracic Surgery, Jining No. 1 People’s Hospital, Jining, Shandong, China; ^2^ Institute of Thoracic Surgery, Jining Institute of Medical Sciences, Jining, Shandong, China; ^3^ School of Clinical Medicine, Jining Medical University, Jining, Shandong, China; ^4^ Department of Thoracic Surgery, Huzhou Central Hospital, Huzhou, Zhejiang, China

**Keywords:** Neoadjuvant, sintilimab, esophageal squamous cell carcinoma, Immunotherapy, overall survival

## Abstract

**Background:**

The study was conducted in order to investigate whether neoadjuvant immunotherapy combined with chemotherapy can bring survival benefits to patients with locally advanced resectable esophageal squamous cell carcinoma (ESCC) in the real world.

**Methods:**

We retrospectively analysed patients with locally advanced resectable ESCC who underwent surgery at the Jining First People’s Hospital from April 2020 to April 2022. Based on their medical history, the enrolled patients were divided into a neoadjuvant immunochemotherapy plus surgery group (nICT group) and a surgery-only group (S group). Primary endpoints were the two-year overall survival (OS) and disease-free survival (DFS) rates. Secondary endpoints were the safety and efficacy of neoadjuvant immunochemotherapy for patients with locally advanced esophageal cancer, and compared the surgery and postoperative outcomes between the two groups.

**Results:**

A total of 47 patients in the nICT group and 73 patients in the S group were included for further analysis, the stage of the nICT group was more advanced than that of the S group. In the group nICT, 8 patients (17%) achieved the complete pathological response (pCR), 29 patients (61.7%) achieved major pathological response (MPR), including 6 patients (12.8%) with a primary tumor achieving pCR but had residual tumor cells in the lymph nodes (pT0N+), and the treatment-related AES was manageable. The surgery and postoperative outcomes were comparable in both groups. The two-year OS and DFS rates for the nICT group were 91.5% and 85.5% respectively, while those for the S group were 71.2% and 68.5%, and Kaplan–Meier survival analysis and log-rank test revealed significant differences in DFS and OS between the two groups. Patients who achieved MPR in the nICT group showed better DFS and OS, while the Three-cycle subgroup did not exhibit any survival benefit compared to the Two-cycle subgroup.

**Conclusions:**

Neoadjuvant sintilimab combined with chemotherapy has promising efficacy and safety in the treatment of locally advanced resectable ESCC. The treatment modality has the potential to become a standard therapy for locally advanced resectable ESCC.

## Introduction

Esophageal cancer (EC) has become one of the common malignant tumours in the world. According to the global cancer statistics in 2022, the number of new patients with EC reached 511,000 and the number of deaths reached 445,000 (1). China is an area with a high incidence of EC. Although the incidence and mortality of EC in China are declining, they are still the main malignant tumours threatening the health of Chinese residents. According to the estimation of the prevalence of malignant tumours in China in 2015, the incidence and mortality of EC ranked sixth and fourth among all malignant tumours respectively (2). In China, more than 90% of EC is esophageal squamous cell carcinoma (ESCC). The simple surgical treatment of EC has low OS and high recurrence rate in five years (3–5). A clinical trial in Japan (JCOG9907) confirmed that neoadjuvant chemotherapy (NCT) can significantly improve the five-year survival rate (55% vs 43%) of resectable stage II and III EC compared with postoperative adjuvant chemotherapy (6). The 5010 study in China showed that patients treated with neoadjuvant chemoradiotherapy (NCRT) have a five-year survival rate of 60% (7). The ten-year OS rate of CROSS study showed that the neoadjuvant radiotherapy and chemotherapy plus surgery group had an OS of 38%, while the surgery alone group had an OS of 25%, and the risk of death was reduced by 40% (8). However, 40%–50% of patients still have tumour recurrence, and distant metastasis is the most common (8, 9). In general, NCRT or NCT combined with surgical resection can improve the survival rate of patients with locally advanced resectable EC, but the prognosis of this group of patients is still not ideal (10). It is still necessary to explore more effective modes of treatment for EC so as to improve the long-term survival of EC patients and reduce recurrence and metastasis.

In recent years, PD-1 inhibitor has become a highly popular immunotherapy for cancer. By blocking the interaction between PD-1 and PD-L1, it can restore the anti-tumour activity of T lymphocytes, enhance the immune response and reduce the proliferation and metastasis of tumour cells. With the breakthrough progress of PD-1 inhibitor and PD-L1 inhibitor in the immunotherapy of melanoma, non-small cell lung cancer, kidney cancer and other tumours, the research on EC was gradually launched, and initial results were achieved (11). At present, immunotherapy for advanced EC has moved from the second line to the first line (12–14). Immunochemotherapy has become the standard first-line treatment for advanced EC, which can bring significant survival benefits to patients (14). Whether neoadjuvant immunochemotherapy can bring more opportunities to cure patients with locally advanced resectable EC has become a key research direction of EC.

Currently, multiple clinical studies on neoadjuvant immunotherapy for EC are being conducted, and they have achieved surprising results (15–17). Sintilimab is a fully humanised recombinant IgG4 monoclonal antibody targeting PD-1 (18). It has a relatively low treatment cost and is considered safe and effective in clinical settings. While multiple studies have affirmed the clinical efficacy of neoadjuvant sintilimab in the treatment of EC (19–21), data on its effectiveness and safety in real-world settings remains insufficient. Currently, clinicians still have concerns about the efficacy and safety of neoadjuvant immunochemotherapy, such as whether it increases the risk of disease progression, whether treatment-related adverse reactions are controllable, whether it increases the difficulty of surgery or increases the risk of postoperative complications, and it is crucial that there is still lack of long-term follow-up results. These clinical questions require further exploration, and thus, we have designed and conducted the current study.

In this study, we conducted a real-world retrospective analysis of 120 ESCC patients who received neoadjuvant immunochemotherapy (nICT) followed by surgery or surgery alone. The primary aim was to evaluate whether neoadjuvant immunotherapy combined with chemotherapy can bring survival benefits to patients with locally advanced ESCC in the real world.

## Methods

### Patients

This retrospective analysis was conducted on the patients with ESCC who received surgical treatment in Jining First People’s Hospital from April 2020 to April 2022. The study has been reviewed and approved by the Medical Ethics Committee of Jining First People’s Hospital.

The inclusion criteria were: the diagnosis of ESCC by pathological biopsy under electronic gastroscope; cTNM staging; cT2-4aN0-2M0 (8th edition of International Union for Cancer Control TNM classification); being without dysfunction of important organs such as heart, brain, lung, liver and kidney and without other serious diseases before treatment; an ECOG score of 0–1; if the patient is receiving preoperative neoadjuvant therapy, the neoadjuvant therapy scheme is combination chemotherapy with sintilimab.

The exclusion criteria were: age less than 18 years or greater than 80 years; the cancer is combined with other malignant tumours; the patient has received neoadjuvant chemotherapy or neoadjuvant radiochemotherapy; the patient has a history of intraperitoneal surgery, intrathoracic surgery and esophageal surgery; the patient cannot tolerate one-lung ventilation; the patient has an immunodeficiency disease, infectious disease, or another serious systemic disease requiring systemic immunosuppressive treatment.

All enrolled patients were divided into a neoadjuvant immunochemotherapy plus surgery group (nICT group) and a surgery-only group (S group) based on their treatment plan.

### Treatment regimen

All enrolled patients underwent enhanced CT scans of the neck, chest and abdomen as well as neck ultrasound to determine their clinical staging. In addition, position emission tomography (PET) was prescribed when necessary. Patients received the above examinations every two cycles before esophagectomy in the nICT group.

The doses of sintilimab administered were fixed doses of 200 mg every three weeks. The adjuvant chemotherapy regimens included a combination of cisplatin (80mg/m^2^) and albumin-bound paclitaxel (200mg/m^2^) or a single drug regimen of albumin paclitaxel every three weeks. The chemotherapy regimens and doses adjusted by patients’ general condition. The number of neoadjuvant treatment cycles was between two and four.

Patients in the nICT group underwent esophagectomy four to six weeks after neoadjuvant immunochemotherapy. The minimally invasive McKeown esophagectomy was the primary surgical approach in two groups. Ivor Lewis esophagectomy and Sweet esophagectomy were performed on some patients who were expected to have challenges in surgery. Under difficult circumstances, minimally invasive surgery could be transferred to thoracotomy. Enlarged two-field lymphadenectomy was regularly conducted, and standard three-field lymphadenectomy was performed in patients with suspected swollen lymph nodes in the neck. Gastric tubes were used for the reconstruction of digestive tract. All operations were conducted by experienced surgeons with more than 50 cases annually, which ensured the surgery’s quality.

Postoperative adjuvant treatment was administered according to postoperative pathological results, the recovery condition of each patient and treatment-related adverse events (TRAEs). Multidisciplinary team discussion was also necessary.

### Endpoints and assessments

Primary endpoints were the two-year OS and DFS rates. Secondary endpoints were the safety and efficacy of neoadjuvant immunochemotherapy and the following surgery, including: Treatment-related adverse events (TRAEs) during neoadjuvant therapy, complete pathological response (pCR) rate and major pathological response (MPR) rate in the nICT group, and the surgery and postoperative outcomes were compared between the two groups. In addition, we conducted an exploratory analysis to determine whether patients in the nICT group who achieved MPR or who had undergone the three-cycle neoadjuvant therapy had better survival benefits.

Follow-up were conducted through outpatient clinics or phone calls. The follow-up time for patients in the nICT group was calculated from the date of receiving immunochemotherapy, while for the S group, it was calculated from the date of surgery completion. All patients in the two groups were followed up with every three months in the first two years and every six months in the third year after the operation. The content of the follow-up included the patient’s general condition, recent re-examination results and other information. The deadline for the follow-up visits was May 2024.

Patient demographics, treatment pattern, intraoperative outcomes and postoperative complications such as anastomotic leakage, pleural effusion, pulmonary infection and ICU admission and 30-day mortality were recorded. Treatment-related adverse events (TRAEs) were collected from each patient’s medical records. TRAEs were graded according to the Common Terminology Criteria for Adverse Events (CTCAE) version 5.0 of the US National Cancer Institute. Evaluate lymph node response according to the Response Evaluation Criteria in Solid Tumors version 1.1 (RECIST 1.1), and assess esophageal lesion relief through esophageal barium swallowing radiography before and after neoadjuvant treatment, including complete remission (CR), partial remission (PR), disease progression (PD) and disease stability (SD). Finally, a comprehensive assessment was conducted. (The specific evaluation details see **Supplementary Material**). Objective response rate (ORR) was defined as the proportion of patients who achieved CR or PR and disease control rate (DCR) was defined as the proportion of patients who achieved CR, PR, or SD. PCR is defined as the absence of evidence of tumour cells in the surgical specimen, including lymph nodes. MPR is defined as the presence of ≤10% tumour cells in the surgical specimen. OS is defined as the time from the start of follow-up to the date of death from any cause or the date of the last follow-up. DFS is defined as the time from the start of follow-up to the date of disease recurrence or death from any cause.

### Statistical analysis

The continuous variables were expressed as median (quartile difference) and the categorical variables were expressed as numbers (percentage). Differences in categorical variables and continuous variables were compared with the x^2^ test or Fisher’s exact test and t-test respectively. OS and DFS were calculated using the Kaplan–Meier method and were then compared by the log-rank test. Statistical analyses were performed using SPSS 27.0 (IBM Corp.) and R (v.4.4.0). P<0.05 (two-sided) was considered statistically significant.

## Results

### Patient selection and baseline characteristics

According to the inclusion criteria, a total of 47 patients in the nICT group and 73 patients in the S group were included for further analysis. Baseline demographic and clinicopathological characteristics are summarised in **Table 1**. There were no significant differences between the two groups in age, smoking, drinking habits, comorbidities or tumour location. No significant gender difference was detected between the two groups, both in which men were predominant: 72.3% and 82.2%, respectively (p =0.201). There were significant differences between the two groups in the clinical N stage, clinical T stage and clinical TNM stage. In the nICT group, cT4a, cN2 and IVA accounted for 42.6%, 19.1% and 42.6% respectively, and the proportion of cT4a, cN2 and IVA is 19.2%, 1.4% and 19.2% respectively in the S group. Obviously, the stage of the nICT group was more advanced than that of the S group.

**Table 1:** Baseline demographics and clinical characteristics (n=120).

Characteristic	GROUP nICT No. (%)	GROUP S No. (%)	X^2^/t	p
Number	47	73		
Age			-1.624	0.107
Median	65	67		
Range	46–77	49–79		
Gender			1.635	0.201
Male	34 (72.3)	60 (82.2)		
Female	13 (27.7)	13 (17.8)		
Smoker			1.894	0.169
Yes	25 (53.2)	48 (65.8)		
No	22 (46.8)	25 (34.2)		
Drinker			0.798	0.372
Yes	18 (38.3)	34 (46.6)		
No	29 (61.7)	39 (53.4)		
Comorbidities				
Hypertension	13 (27.7)	16 (21.9)	0.514	0.473
Diabetes mellitus	4 (8.5)	5 (6.8)	0.114	0.736
Coronary disease	4 (8.5)	10 (13.7)	0.747	0.388
Tumour location			4.440	0.109
Upper	5 (10.6)	5 (6.8)		
Middle	33 (70.2)	41 (56.2)		
Lower	9 (19.1)	27 (37)		
Clinical T stage			8.391	0.015
cT2	3 (6.4)	11 (15.1)		
cT3	24 (51.1)	48 (65.8)		
cT4a	20 (42.6)	14 (19.2)		
Clinical N stage			12.646	0.002
cN0	16 (34.0)	37 (50.7)		
cN1	22 (46.8)	35 (47.9)		
cN2	9 (19.1)	1 (1.4)		
Clinical stage group			7.791	0.020
II	15 (31.9)	35 (47.9)		
III	12 (25.5)	24 (32.9)		
IVA	20 (42.6)	14 (19.2)		

In the nICT group, all patients received sintilimab combined with chemotherapy, 45 patients received sintilimab combined with albumin-bound paclitaxel and cisplatin and two patients received sintilimab combined with albumin-bound paclitaxel. 33 cases received two cycles and 14 cases received three cycles.

### Effectiveness and safety of the neoadjuvant treatment

Radiographic response evaluation was performed in group nICT before surgery. Of the 47 patients, seven (14.9%) achieved CR, 35 (74.5%) achieved PR and five patients (10.6%) achieved SD. The ORR and DCR were 89.4% and 100%, respectively. Pathological evaluation was conducted after operation. 8 patients (17%) achieved the complete pathological response (pCR), 29 patients (61.7%) achieved major pathological response (MPR), including 6 patients (12.8%) with a primary tumor achieving pCR but had residual tumor cells in the lymph nodes (pT0N+) (**Table 2**).

**Table 2:** Efficacy of neoadjuvant treatment.

Outcomes	No. (%)
Radiographic evaluation	
CR	7 (14.9)
PR	35 (74.5)
SD	5 (10.6)
ORR (%)	42 (89.4)
DCR (%)	47 (100)
Pathological evaluation	
yp T0N0M0 (pCR)	8 (17.0)
yp T0N+M0	6 (12.8)
MPR	29 (61.7)

TRAEs related to drugs before surgery were observed in 42 (89.4%) patients in the nICT group. The common sintilimab-related adverse reactions are skin reactions, including pruritus (11 of 47, 23.4%), rash (22 of 47, 46.8%) and most of them are minor adverse events of grade 1–2. Others were pneumonia (2 of 47, 4.3%) and hypocorticism (2 of 47, 4.3%), among which grade 3–4 adverse events were lower. Adverse reactions related to chemotherapy included common haematological toxicity. Gastrointestinal discomfort and alopecia also had a high incidence, but most of them were grade 1–2 adverse events. Grade 3–4 adverse events were leukopenia in three cases (6.4%) and thrombocytopenia in one case (2.1%) (**Table 3**).

**Table 3:** Treatment-related adverse events (TRAEs) based on CTCAE version 5.0.

Adverse events	Grades 1–2, No. (%)	Grades 3–4, No. (%)
Leukopenia	15 (31.9)	3 (6.4)
Thrombocytopenia	12 (25.5)	1 (2.1)
Anemia	14 (29.8)	0
Pruritu	11 (23.4)	0
Rash	22 (46.8)	0
Alopecia	39 (83.0)	0
Nausea and vomiting	31 (66.0)	0
Pneumonia	2 (4.3)	0
Joint and muscle pain	27 (57.4)	0
Liver injury	1 (2.1)	0
Cardiac involvement	0	1 (2.1)
Hyperglycaemia	0	1 (2.1)
Hypocorticism	1 (2.1)	1 (2.1)
Hypothyroidism	3 (6.4)	0

### Surgery and postoperative safety

No surgery was delayed beyond six weeks after the final neoadjuvant therapy in group nICT. The percentages of minimally invasive esophagectomy (MIE) were 95.7% and 95.9% in the nICT group and group S respectively, while one case in each group was converted to thoracotomy (p = 0.632). Radical resection (R0) was achieved in 46 (97.9%) and 71 (97.3%) patients in the nICT group and group S respectively (p=0.834). No exploratory surgery was performed in either group. No significant difference was found between the two groups in the operation time (p=0.787), intraoperative blood loss (p=0.258), postoperative hospital stay (p=0.314) or lymph node dissection number (p=0.270) (**Table 4**).

**Table 4:** Surgery and postoperative outcomes.

Characteristic	GROUP nICT No. (%)	GROUP S No. (%)	X^2^/t	p
Surgery approach			0.002	0.653
MIE	45(95.7)	70(95.9)		
Thoractomy esophagectomy	2(4.3)	3(4.1)		
Converted to thoractomy	1(2.1)	1(1.4)	0.100	0.632
R0 resection	46(97.9)	71(97.3)	0.044	0.834
Operation time			0.270	0.787
Median	246	240		
Range	180–420	170–355		
Bleeding			1.143	0.258
Median	150	150		
Range	50–800	20–300		
Lymph node dissection number			-1.108	0.270
Median	26	30		
Range	9–58	14–71		
Postoperative hospital stay			-1.011	0.314
Median	11	11		
Range	9–45	9–55		
Surgical complications				
Hoarseness	8 (17.0)	12 (16.4)	0.007	0.993
Anastomotic fistula	3 (6.4)	6 (8.2)	0.139	0.709
Chylothorax	1 (2.1)	1 (1.4)	0.100	0.752
Pulmonary Infections	15 (31.9)	18 (24.7)	0.755	0.385
Arrhythmia	1 (2.1)	4 (5.5)	0.804	0.370
Pleural effusion	4 (8.5)	6 (8.2)	0.003	0.955
Pneumothorax	2 (4.3)	2 (2.7)	0.204	0.652
ICU admission	0	4(5.5)	0.154	0.132
30-day mortality	0	2(2.7)	0.519	0.368

Postoperative surgical complications within 30 days are summarised in **Table 4**. The incidence of hoarseness, anastomotic fistula, tracheoesophageal fistula, pulmonary infections, arrhythmia, pleural effusion, pneumothorax and chylothorax were comparable in both groups. One case of chylothorax in each group was successfully treated by conservative treatment. Four cases of ICU admission and two cases of 30-day mortality were observed in the S group, while no ICU admission and no 30-day mortality occurred in the nICT group. The causes of death of the two patients in the S group were tracheoesophageal fistula and pneumonia.

### Follow-up

**Figure 1:**
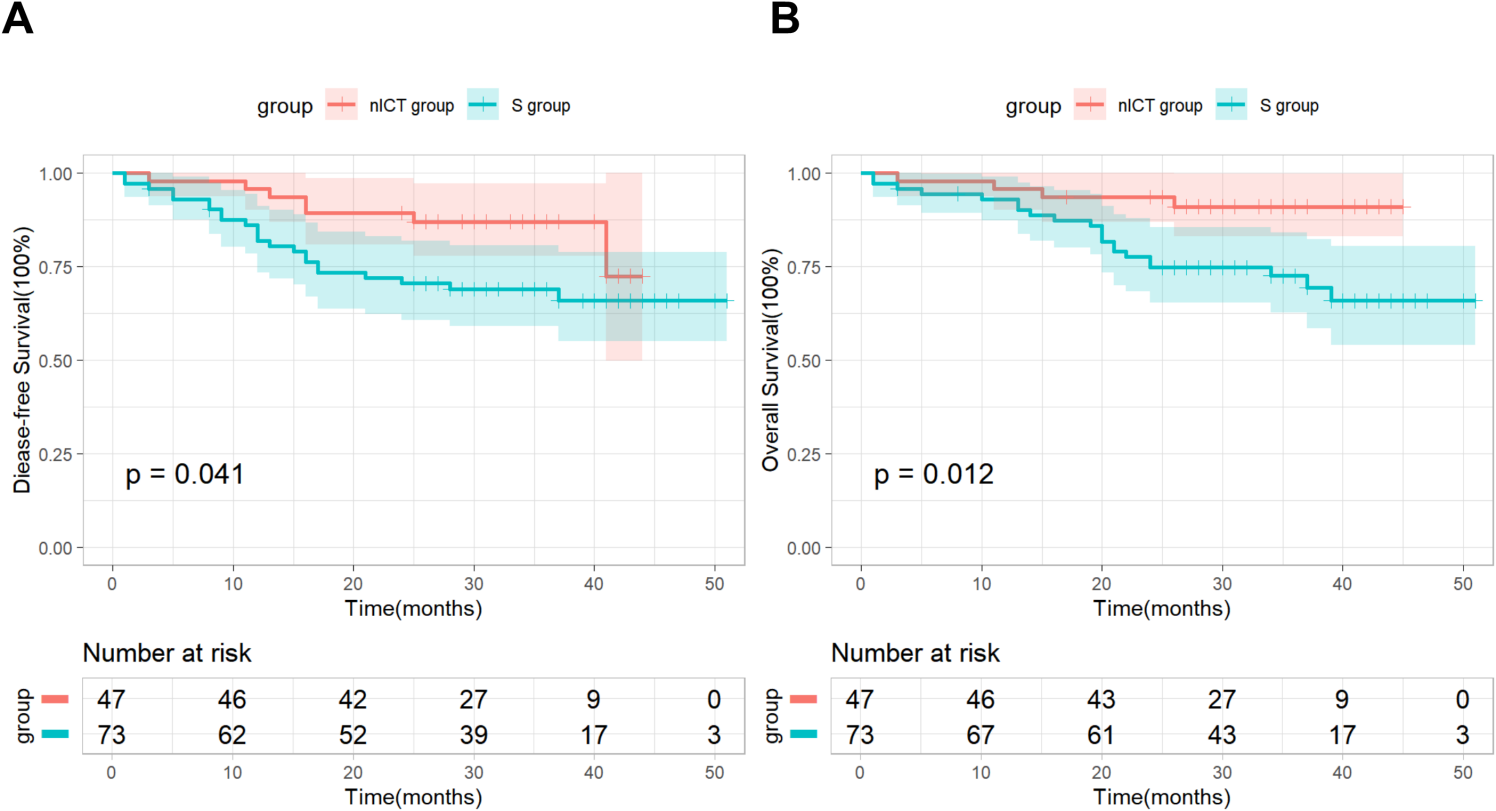
DFS **(A)** and OS **(B)** between the nICT group and the S group.

As of the cut-off date of May 2024, all patients had been followed up with for more than two years. The median follow-up time was 31 months (interquartile range: 27.0–37.0) in the nICT group, while the median follow-up time was 34 months (interquartile range: 29.5–42.0) in the S group. The median OS and DFS have not yet been reached in both groups. Specifically, the two-year OS and DFS rates for the nICT group were 91.5% and 85.5%, while the two-year OS and DFS rates for the S group were 71.2% and 68.5%. Kaplan–Meier survival analysis and log-rank test revealed significant differences in DFS and OS between the two groups (**Figure 1**).

**Figure 2:**
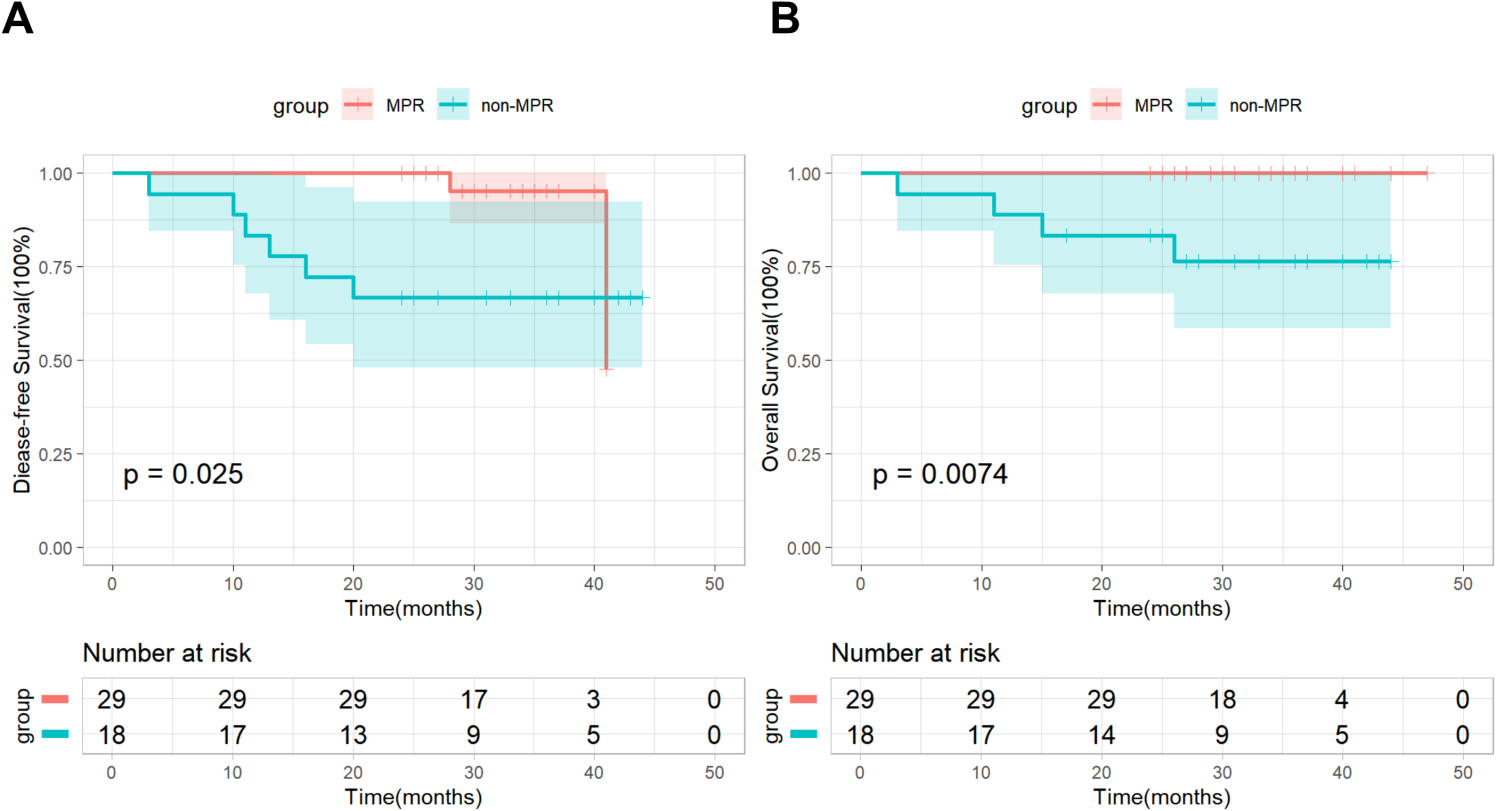
DFS **(A)** and OS **(B)** between the MPR subgroup and the non-MPR subgroup.

**Figure 3:**
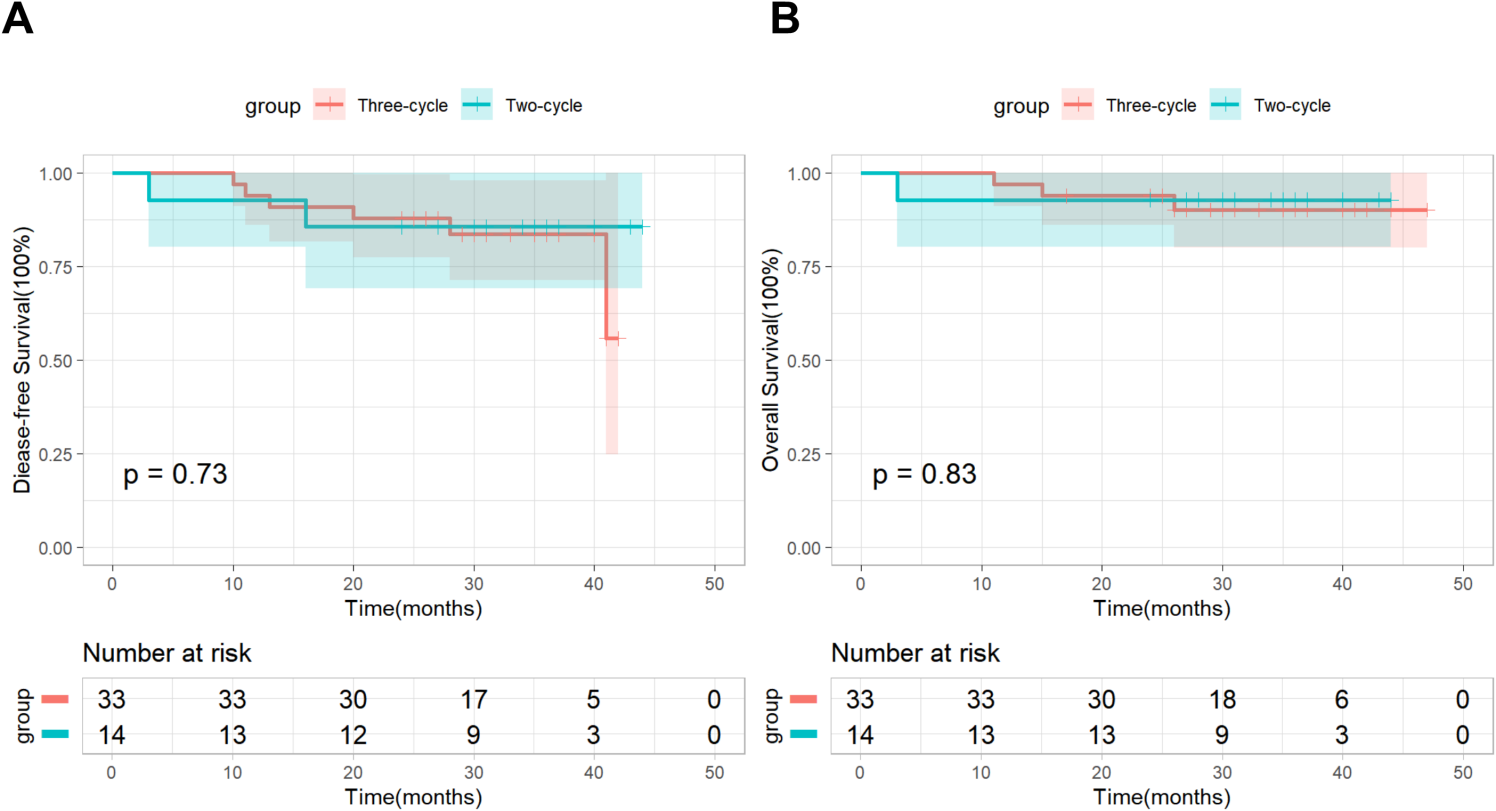
DFS **(A)** and OS **(B)** between the Three-cycle subgroup and the Two-cycle subgroup.

In addition, based on postoperative pathological results, we conducted a subgroup analysis by dividing patients in the nICT group into an MPR subgroup (n=29) and a non-MPR subgroup (n=18). As of the follow-up cutoff date, all patients (100%) in the MPR subgroup were still alive, and recurrence was observed in only 2 (6.9%) patients, Kaplan-Meier survival analysis combined with the log-rank test demonstrated significant differences in both DFS and OS between the two subgroups (**Figure 2**). Based on the number of neoadjuvant therapy cycles, patients in the nICT group were divided into a Two-cycle subgroup (n=33) and a Three-cycle subgroup (n=14), the Kaplan–Meier survival analysis and log-rank test revealed no significant differences in DFS and OS between the two subgroups (**Figure 3**).

## Discussion

Although surgery following neoadjuvant chemoradiotherapy represents the standard treatment protocol for locally advanced ESCC, neoadjuvant radiotherapy may induce edema and fibrosis in the tissues surrounding the tumor, thereby elevating the surgical complexity. Furthermore, it may also augment the risk of postoperative bleeding and increase fluid leakage in patients. Consequently, the proportion of preoperative concurrent chemoradiotherapy conducted in China remains relatively low (22). Additionally, findings from several clinical trials and retrospective studies indicate that the clinical efficacy of neoadjuvant chemotherapy falls significantly short of that of neoadjuvant chemoradiotherapy (23, 24). The treatment of neoadjuvant immunochemotherapy followed by surgery brought more chances to cure patients with locally advanced resectable EC, which has become the focus of many studies on locally advanced EC. Most of these studies are single-arm, small-sample clinical studies, while other large prospective randomised controlled clinical studies have not yet yielded results, and there is no definitive conclusion regarding whether there is a long-term survival benefit. Therefore, real-world evidence can also serve as a good reference. Thus, we conducted this real-world retrospective study to investigate the efficacy, safety and the two-year follow-up results of neoadjuvant sintilimab combined with chemotherapy for locally advanced resectable ESCC. In the study that enrolled a relatively large population, several encouraging results were identified, which are consistent with previous studies.

Compared to the S group, the proportion of cT4a and cN2 patients was significantly higher in the nICT group, and the stage of the nICT group was more advanced than that of the S group. However, the surgical data did not reflect that the patients in the nICT group had advanced tumours. Compared to the S group, the percentages of MIE, rates of conversion to thoracotomy and radical resection rates did not increase in the nICT group. No significant difference was found between the two groups in the operation time, intraoperative blood loss, postoperative hospital stay or lymph node dissection number. In the study, a median of 26(9 to 58) lymph nodes were dissected in the nICT group, while in NEOCRTEC5010 study, the median lymph node dissection number was 20(15 to 27) in nCRT group (25). The R0 resection rate was 97.9% (46/47) in the nICT group, which is comparable to the NEOCRTEC5010 study (25). In our surgical practice, most patients in the nICT group had no obvious fibrosis or dense adhesions in the esophageal bed. These findings suggest that the neoadjuvant sintilimab combined with chemotherapy effectively downstaged the tumour and reduced the difficulty of surgery.

The postoperative outcomes shows that the incidence of hoarseness, anastomotic fistula, tracheoesophageal fistula, pulmonary infections, arrhythmia, pleural effusion, pneumothorax and chylothorax were comparable in both groups. The incidence of anastomotic fistula in the nICT group was 6.4%, which was lower than that previously reported in the CROSS study (22%) (26) and NEOCRTEC5010 study (8.6%) (25). The neoadjuvant immunotherapy did not increase postoperative mortality, which was consistent with the previous reports (27). Two cases of 30-day mortality were observed in the S group, and the causes of death were tracheoesophageal fistula and pneumonia, which may be related to severe tumour invasion and long operation time. In general, neoadjuvant sintilimab combined with chemotherapy was well-tolerated and safe. The neoadjuvant immunochemotherapy should be prescribed to more patients with locally advanced resectable ESCC.

TRAEs related to drugs before surgery were observed in 42 (89.4%) patients in the nICT group, and TRAEs of grade 3–4 were observed in 14.9% of patients, which is significantly lower than the 54.3% reported in the NEOCRTEC5010 nCRT group (25). In the study, the adverse reactions related to sintilimab were consistent with those reported in previous studies (21).

Recently, a number of small-sample clinical trial of neoadjuvant immunotherapy combined with chemotherapy for locally advanced EC have been published successively. The pCR rate of each study is between 18.8% and 50%, and the MPR rate is 43.8%–72.4% (28–35). In the study, the pCR rate was 17%, the reason for the low pCR rate in this study may be that many patients had advanced tumours and that most of the patients received two cycles of neoadjuvant therapy. Adding neoadjuvant therapy cycles may increase the pCR rate, but it may also increase adverse drug reactions and surgical complications. It is noteworthy that the MPR rate was 61.7% in this study, including 6 patients (12.8%) with a primary tumor achieving pCR but had residual tumor cells in the lymph nodes (pT0N+), which is comparable to that in other clinical trials, indicating a good response of locally advanced resectable ESCC to neoadjuvant sintilimab.

Consistent with previous studies demonstrating the survival benefits of neoadjuvant immunotherapy in EC (15, 36, 37), our study found significant differences in OS and DFS between the two groups after more than two years of follow-up. In the study, the 2-year OS and DFS rates for the nICT group were 91.5% and 85.5%, which is close to the results of the PERFECT study (2-year OS and PFS rates was 92%, 85%) (38) and far higher than the SCALE-1 study (2-year OS and PFS rates was 78%, 63.8%) (16), indicating that reducing radiotherapy in neoadjuvant therapy regimen for ESCC may also achieve good survival benefit. In a Phase II clinical trial on neoadjuvant camrelizumab combined with chemotherapy for locally advanced ESCC, the 2-year OS rate and DFS rate were 97.6% and 92.3%, respectively (39). The reason for these higher rates compared to our results may be that 95.7% of the patients in that study received three cycles of neoadjuvant therapy and all patients were clinically staged as II-III. However,only 14 patients (29.8%) received three cycles of neoadjuvant therapy, and 42.6% of the patients were clinically staged as IVA in our study. Furthermore, there may be variations in the efficacy of different neoadjuvant drugs. In the study, patients in the Three-cycle subgroup of the nICT group did not demonstrate significant survival benefits compared to the Two-cycle subgroup, which could potentially be attributed to the relatively small number of patients who underwent the Three-cycle neoadjuvant therapy. Whether increasing the number of neoadjuvant therapy cycles would enhance survival benefits remains unknown, and this is a direction that requires further research. Recently, the NICE study (40) released the two-year follow-up results of neoadjuvant camrelizumab combined with chemotherapy for clinical N2-3 ESCC: the study showed that distant recurrence remains the primary recurrence pattern, and MPR may be associated with a lower recurrence rate and better survival, in addition, the 2-year OS and RFS rates were 78.1% and 67.9%, respectively. The recurrence pattern in the nICT group of this study is consistent with the NICE study: among the 6 patients who recurred, 4 had distant recurrence and 2 had local recurrence. Compared to the NICE study, the study showed better survival benefits, which may be due to the following reasons: all patients enrolled in the NICE study were stage III-IV, while 31.9% of the patients in this study were stage II; there are differences in neoadjuvant treatment regimens and adjuvant treatment strategies. In the study, patients in the nICT group who achieved MPR also demonstrated better survival benefits, this suggests that MPR could potentially serve as an effective indicator for predicting the long-term prognosis of locally advanced resectable ESCC treated with neoadjuvant immunotherapy combined with chemotherapy. Furthermore, all patients in the MPR subgroup survived, regardless of whether they achieved pCR or not, and only two patients who did not achieve pCR experienced recurrence, this might suggest that ESCC patients can derive significant survival benefits as long as they achieve MPR after neoadjuvant immunotherapy (even if pCR is not attained). It is worth noting that postoperative adjuvant therapy in the study was not mandatory and was administered based on factors such as the patient’s pathological results, tolerance to chemotherapy drugs, the patient compliance and other considerations. In the study, ten patients (21.3%) in the nICT group received adjuvant immunochemotherapy, 2 (4.3%) received adjuvant radiotherapy, and 1 (2.1%) received adjuvant chemoradiotherapy after surgery. In the S group, 19 patients (26.0%) received adjuvant chemotherapy and 4 (5.6%) received adjuvant radiotherapy after surgery. The postoperative adjuvant therapy in both groups may have also influenced the survival outcomes of the study, but there is a significant discrepancy in postoperative schemes, which cannot be compared for analysis. Of course, our findings need to be validated through larger randomized controlled trials and further follow-up. In conclusion, neoadjuvant immunotherapy combined with chemotherapy for locally advanced ESCC has shown remarkable survival benefits, and long-term results still require further follow-up.

However, there are also some limitations: (1) the patients enrolled in this study were all from a single centre, and our results need to be further validated through multi-centre, randomised controlled trials with a larger sample size; (2) this study did not further explore relevant tumour markers, whose expression levels may affect treatment outcomes.

## Conclusions

Neoadjuvant sintilimab combined with chemotherapy has promising efficacy and safety in the treatment of locally advanced resectable ESCC, and during the follow-up period of over two years, it exhibited significant survival benefits, this treatment modality has the potential to become a standard therapy for locally advanced resectable ESCC.

## Data Availability

The raw data supporting the conclusions of this article will be made available by the authors, without undue reservation.
