# Supplementary material for: Mid-term follow-up results of neoadjuvant sintilimab combined with chemotherapy for locally advanced resectable esophageal squamous cell carcinoma
Source: Front Immunol. 2024 Dec 4;15:1453176. doi: 10.3389/fimmu.2024.1453176 (PMC11652524; doi:10.3389/fimmu.2024.1453176)
Supplement: Supplementary file 1 [file Table1.docx]

Specific radiological response assessment details

1. lymph node (target lesions)

Evaluation standard (RECIST 1.1)

Comparison with baseline chest CT:

| Complete Response (CR) | All lesions disappeared. |
| --- | --- |
| Partial Remission (PR) | The sum of the longest diameter of lesions decreased by more than or equal to 30%. |
| Stable Disease (SD) | The sum of the longest diameter of lesions does not meet the evaluation criteria of disease progression and partial remission. |
| Progressive Disease (PD) | The sum of the longest diameter of lesions increased by 20% or more. |

1. Esophageal lesion (non-target lesions)

Evaluation standard (Modified from "Efficacy Evaluation Criteria of Esophageal Barium Swallowing Radiography at the End of Radiotherapy for Esophageal Cancer in Japan")

| Complete Response (CR) | The lesion disappeared completely, the esophageal wall was soft, and no tumor remained. |
| --- | --- |
| Partial Remission (PR) | Along the long axis of the esophagus, the length of the lesion subsided by more than 50%, but not completely, and the esophageal lumen stenosis improved. |
| Stable Disease (SD) | In the long axis of the esophagus, the length of the lesion was less than 50%, or regardless of tumor changes, lumen stenosis of the esophagus is evident but does not progress. |
| Progressive Disease (PD) | The length of lesions increased by more than 20%, and esophageal lumen stenosis was more obvious than before. |

1. Comprehensive evaluation：

| lymph node  Esophageal lesion | CR | PR | SD | PD |
| --- | --- | --- | --- | --- |
| CR | CR | PR | SD | PD |
| PR | PR | PR | SD | PD |
| SD | SD | SD | SD | PD |
| PD | PD | PD | PD | PD |
